# Age-related neutrophil activation in Hermansky-Pudlak Syndrome Type-1

**DOI:** 10.1186/s13023-025-03758-5

**Published:** 2025-05-12

**Authors:** Lourdes Marinna Caro-Rivera, Sonya Malavez-Cajigas, Mercedes Lacourt-Ventura, Andrea P. Rivera-Torres, Dorca E. Marcano-Jiménez, Pablo López-Colon, José Muñiz-Hernández, Enid Rivera-Jiménez, Mónica Egozcue-Dionisi, Rosa Román-Carlo, Wilfredo De Jesús-Rojas, Marcos J. Ramos-Benítez

**Affiliations:** 1 Department of Basic Sciences, Ponce Health Sciences University and Ponce Research Institute, Ponce, Puerto Rico; 2 Department of Medicine, San Juan Bautista School of Medicine, Caguas, Puerto Rico; 3 Department of Pediatrics, Medical Sciences Campus, University of Puerto Rico, San Juan, Puerto Rico

## Abstract

Hermansky-Pudlak Syndrome (HPS) type 1 (HPS-1) is an autosomal recessive disorder characterized by oculocutaneous albinism, platelet dysfunction, and pulmonary fibrosis (HPS-PF), the leading cause of mortality in these patients. HPS-PF manifests earlier than idiopathic pulmonary fibrosis, typically between 30 and 40 years of age. The etiology and drivers of HPS-PF progression remain poorly understood, and no FDA-approved therapies exist. Neutrophil extracellular traps (NETs) and neutrophil-derived mediators have emerged as key players in fibrosis, promoting lung injury, inflammation, and fibroblast activation. This study evaluates the role of neutrophil activation in age-related changes in patients with HPS-1, focusing on differences in inflammatory markers, neutrophil granules, and NETosis capacity. We observed significantly elevated levels of NETs, neutrophil granule proteins (NE, NGAL, LF), and inflammatory cytokines (IL-8, IL-6) in patients with HPS-1 older than 40 years compared to younger patients and healthy controls. Additionally, fibrosis-related markers (MMP-7 and MMP-8) were significantly higher in older patients. Elevated levels of anandamide (AEA), a circulating marker of HPS-PF, were positively associated with neutrophil granule markers in older patients, suggesting its association with fibrosis. Neutrophils from older patients also demonstrated increased NETosis capacity. These findings suggest that age-related neutrophil activation may contribute to an inflammatory environment that promotes fibrosis progression in HPS-1.

**Supplementary Information:**

The online version contains supplementary material available at 10.1186/s13023-025-03758-5.

## Introduction

Hermansky-Pudlak Syndrome (HPS) is an autosomal recessive disorder with 11 different subtypes, each characterized by varying degrees of oculocutaneous albinism, platelet storage pool deficiency, and progressive pulmonary fibrosis (PF) [1]. HPS type 1 (HPS-1) is the most prevalent and severe form, leading to the inevitable development of PF and respiratory failure in all affected individuals [2]. HPS-1 is particularly notable for its high prevalence in certain populations, including Puerto Rico, where the incidence is approximately 1 in 1,800 individuals in the northwestern part of the island, accounting for half of the global cases [3]. All patients with HPS-1 develop pulmonary fibrosis (HPS-PF), representing the leading cause of death in this population with less than 10-year life expectancy once the diagnosis of restrictive lung disease is made [4].

HPS-PF is characterized by a progressive fibrogenesis of the lung parenchyma and interalveolar septa that eventually leads to death from respiratory failure [5]. The pulmonary fibrosis in these patients typically manifests at the age of 30–40 years old, this represents 10 to 20 years earlier than other forms, such as idiopathic pulmonary fibrosis (IPF), which typically manifests in individuals over 50 years old [2]. It is extremely important an early diagnosis of HPS-PF as the average survival time after a late diagnosis can be significantly reduced. Lung transplantation remains the only definitive therapy for patients with HPS-PF, though antifibrotics may offer some benefit in select cases. While pulmonary fibrosis is the leading cause of mortality in HPS-1, the mechanisms driving its onset and progression are not well understood.

Neutrophil extracellular traps (NETs) have emerged as key players in the pathogenesis of various chronic lung diseases [6], including pulmonary fibrosis. NETs are web-like structures composed of DNA, histones, and antimicrobial proteins that are released by activated neutrophils to trap and kill pathogens [7]. However, excessive or dysregulated NETs formation can contribute to tissue damage and inflammation [8]. In addition to NETs, neutrophil granules containing enzymes like elastase (NE), neutrophil gelatinase-associated lipocalin (NGAL), and lactoferrin (LF) further promote tissue breakdown and inflammation.

Studies have shown that NETs contribute to the development and progression of pulmonary fibrosis through several mechanisms. NETs promote fibroblast differentiation and function, driving fibrosis in the lungs [9]. They also induce epithelial and endothelial cell death, further exacerbating lung injury [10]​​. Additionally, NETs can activate lung fibroblasts via the TLR9-miR-7-Smad2 pathway, leading to polymyositis-related interstitial lung diseases [11]. In the context of IPF, a study found that elevated levels of NETs markers in bronchoalveolar lavage (BAL) are associated with worse pulmonary function and reduced survival. The study showed that patients with higher NETs levels in BAL had a significant decline in forced vital capacity (FVC) and diffusing capacity for carbon monoxide (DLCO), along with a higher mortality rate over a two-year period [12]. In HPS-PF, a recent case report presented histological evidence of extracellular traps formation in an explanted lungs tissue of a patient with HPS-PF. Providing the first report of the potential role of NETs in disease progression [13].

Since HPS-PF typically manifests around the age of 40, gaining insight into the role of age-related neutrophil activation is highly relevant. This study aims to evaluate the association between neutrophil activation markers and age in patients with HPS-1, providing a deeper understanding of the inflammatory processes that may contribute to fibrosis progression. By investigating neutrophil-derived mediators, cytokines, and NETosis capacity, this study offers valuable insights into potential mechanisms underlying disease progression and identify novel therapeutic targets for HPS-PF.

## Methods

### Participant recruitment

Patients diagnosed with HPS-1 and non-HPS controls were recruited for this study, through the Institutional Review Board (IRB) protocol number: 2303138796. Inclusion criteria for patients with HPS-1 included a confirmed genetic diagnosis and the presence of clinical symptoms consistent with HPS-1. Non-HPS controls were selected based on the absence of any clinical indications of HPS-1. Patients were recruited through the Hermansky-Pudlak Syndrome community clinics at Mayagüez Medical Center in Mayagüez, Puerto Rico. Patients were invited to voluntarily participate and provided consent. Controls were selected to match the average age of patients with HPS-1, ensuring comparability between groups. A median split analysis was used to divide participants into two groups: those aged ≤ 40 years and those aged > 40 years. Demographic information, spirometry data, and pulmonary fibrosis status were collected retrospectively. Pulmonary fibrosis diagnoses were confirmed through CT scan reports from routine clinical care, as interpreted by radiologists at the respective institutions. While fibrosis status was recorded, the primary objective of the study was to assess age-related changes in neutrophil markers with fibrosis markers analyzed as exploratory outcomes. Spirometry data, including Forced Vital Capacity (FVC) and the FEV1/FVC ratio, were obtained from clinical assessments at HPS clinics. Participant demographics are summarized in Table 1.

**Table 1:**
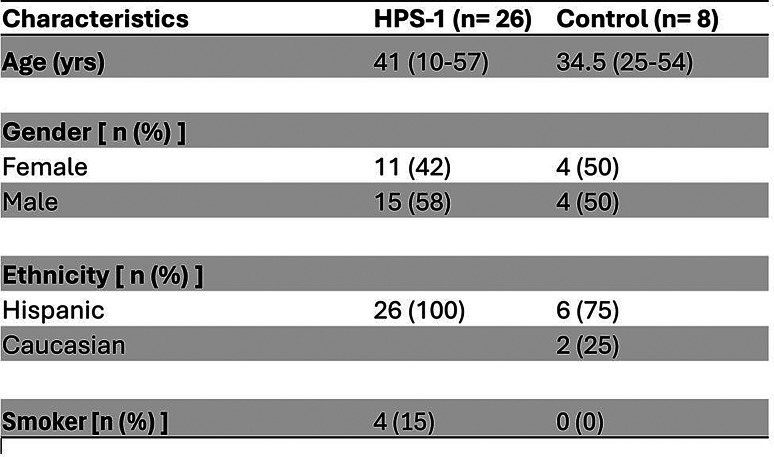
Patients demographics

### Measurement of Circulating NETs and inflammatory markers

Blood samples were collected in EDTA tube and processed to obtain plasma. Levels of cytokines, including G-CSF, IL-12 P70, IL-1β, IL-6, IL-8, and TNF-α, were measured using the Milliplex Human Cytokine/Chemokine/Growth Factor Panel A Magnetic Bead Panel, following the manufacturer’s instructions (Merck KgaA, Germany, Cat. No HCYTA-60 K-07). Levels of granule-derived proteins, including NE, NGAL, and LF, were measured using the Human Sepsis Magnetic Bead Panel 3, following the manufacturer’s instructions (Merck KgaA, Germany, Cat. No. HSP3MAG-63 K-03). Levels of circulating NETs were quantified using the Human Neutrophil Extracellular Traps (NETs) GENLISA ELISA Kit from Krishgen BioSystems, California (Cat. No. KBH4548), according to the manufacturer’s instructions. Additionally, plasma levels of anandamide (AEA) were measured using the Human Anandamide (AEA) ELISA Kit (MyBioSource, California, Cat. No. MBS167217), according to the manufacturer’s protocol. Levels of circulating matrix metalloproteinase including MMP-7, MMP-8, and MMP-9, were measured using the Simple Plex Cartridge Kit (ELLA Technology) following the manufacturer’s instructions (Bio-Techne, Minnesota, Cat. No. SPCKA-CS-011888).

### Live cell imaging

Neutrophils were isolated from whole blood collected in EDTA tubes from a subset of patients with HPS-1 under 40 years old (*n* = 4) and over 40 years (*n* = 6), using the EasySep Direct Human Neutrophil Isolation Kit (Stemcell Technologies, Canada, Cat. No. 19666). Neutrophils (30,000 cells/well) were added to each well in 100 µL of Gibco Opti-MEM (Gibco, New York, Cat. No. 31985070) containing 250 nM Cytotox Green (Sartorious AG, Göttingen, Germany, Cat. No. 4633). Cells were stimulated with PMA (100nM) (Thermo Fisher, Massachusetts, Cat. No. AAJ63916MCR), for 5 h. Plates were placed in the Incucyte SX5 (Sartorius AG, Göttingen, Germany) for live-cell imaging. Phase contrast and green fluorescence images were captured at 20x magnification, with an exposure time of 300 milliseconds for green fluorescence. Images were acquired every 30 min for 6 h, with two images per well from distinct regions. The Incucyte software was trained to differentiate between normal cells and those undergoing DNA release using representative images from non-stimulated and stimulated cells. For analysis, the software applied surface fit background correction with a fluorescence threshold of 2.0 Green Calibrated Units (GCU), edge sensitivity of -25, and a minimum mean intensity of 100. Phase contrast analysis used artificial intelligence (AI) confluence detection. The software automatically analyzed the images and calculated the total integrated green intensity (GCU x µm^2^/ image) per well to quantify DNA release associated with NETosis.

### Statistical analysis

Statistical analyses were performed to evaluate differences in biomarker levels and their associations with clinical parameters. The Kruskal-Wallis test was used for group comparisons of circulating neutrophil activation markers and cytokine levels, with statistical significance denoted as follows: *p < 0.05, **p < 0.01, and ***p < 0.001. Simple linear regression was performed to examine the relationship between specific biomarkers and pulmonary function parameters (FVC, and FEV1/FVC ratio), with significance set at *p* ≤ 0.05. For NETosis capacity, comparisons between patients with HPS-1 and non-HPS controls were assessed using two-way ANOVA with multiple comparisons.

## Results

### Circulating levels of NETs and neutrophil granule proteins in patients with HPS-1

**Fig. 1:**
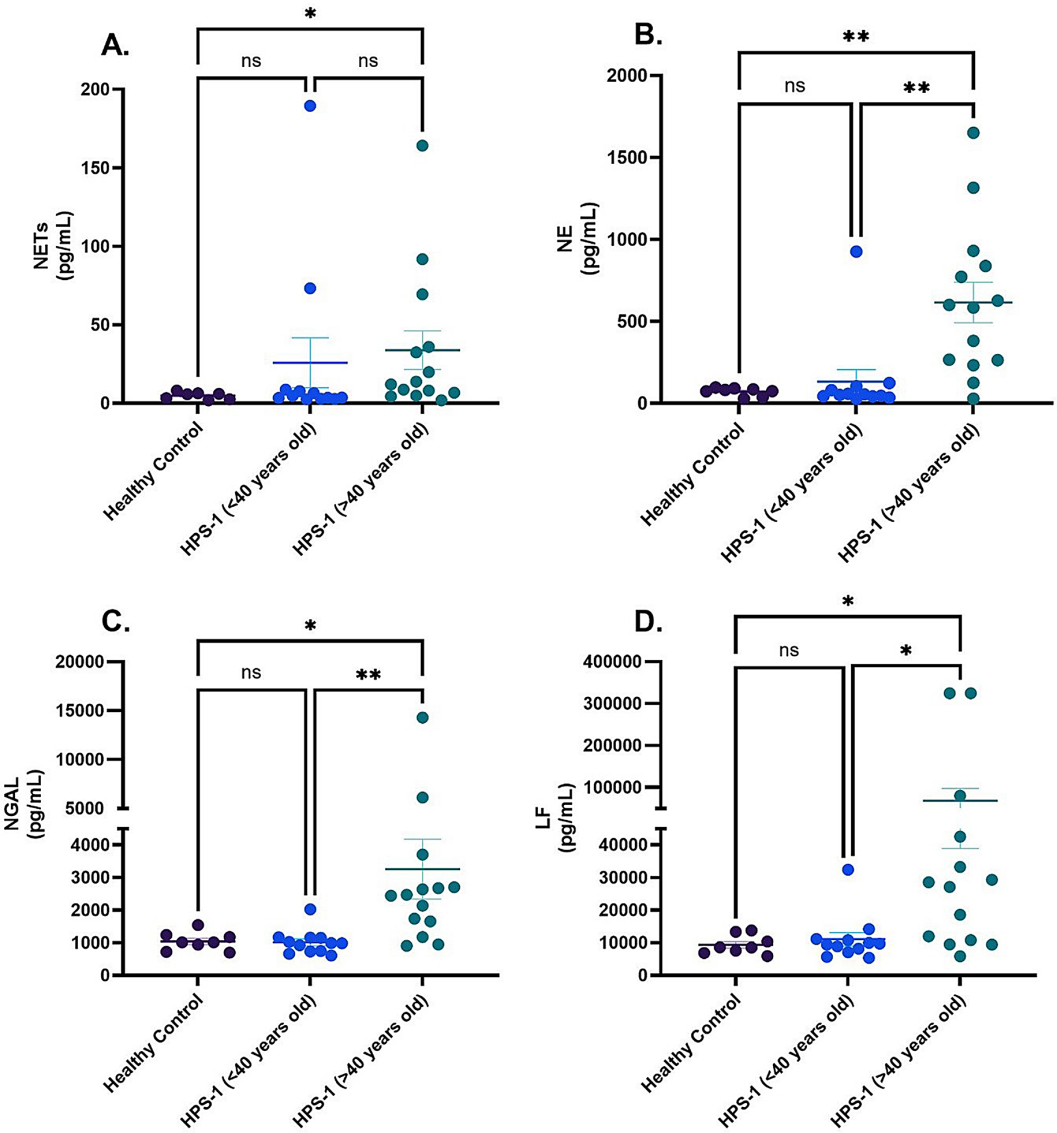
Circulating neutrophil activation markers and NETs in patients with HPS-1. The biomarkers shown are **A**) NETs, **B**) NE, **C**) NGAL, and **D**) LF. Each data point represents an individual patient measurement within the group, with horizontal lines indicating the mean and error bars representing the standard error. The Kruskal-Wallis test was used to compare groups, with statistical significance indicated by asterisks: * for *p* < 0.05, ** for *p* < 0.01, and *** for *p* < 0.001

We evaluated circulating levels of NETs and neutrophil granule-derived proteins, including NE, NGAL, and LF, in patients with HPS-1 (*n* = 26). A median age split of 40 years was applied, dividing participants into two groups: those aged ≤ 40 years and those aged > 40 years, consistent with the age-related onset and progression of HPS-PF. These circulating NETs levels were significantly elevated in patients with HPS-1 over 40 years old only when compared to healthy controls (*P* = 0.04) Fig. 1A. Additionally, levels of neutrophil-derived granules, including NE, NGAL, and LF were elevated in patients over 40 years old compared to both healthy controls and HPS-1 individuals under 40 (*P* > 0.05), Fig. 1B–D.

### Circulating levels of inflammatory markers in patients with HPS-1

**Fig. 2:**
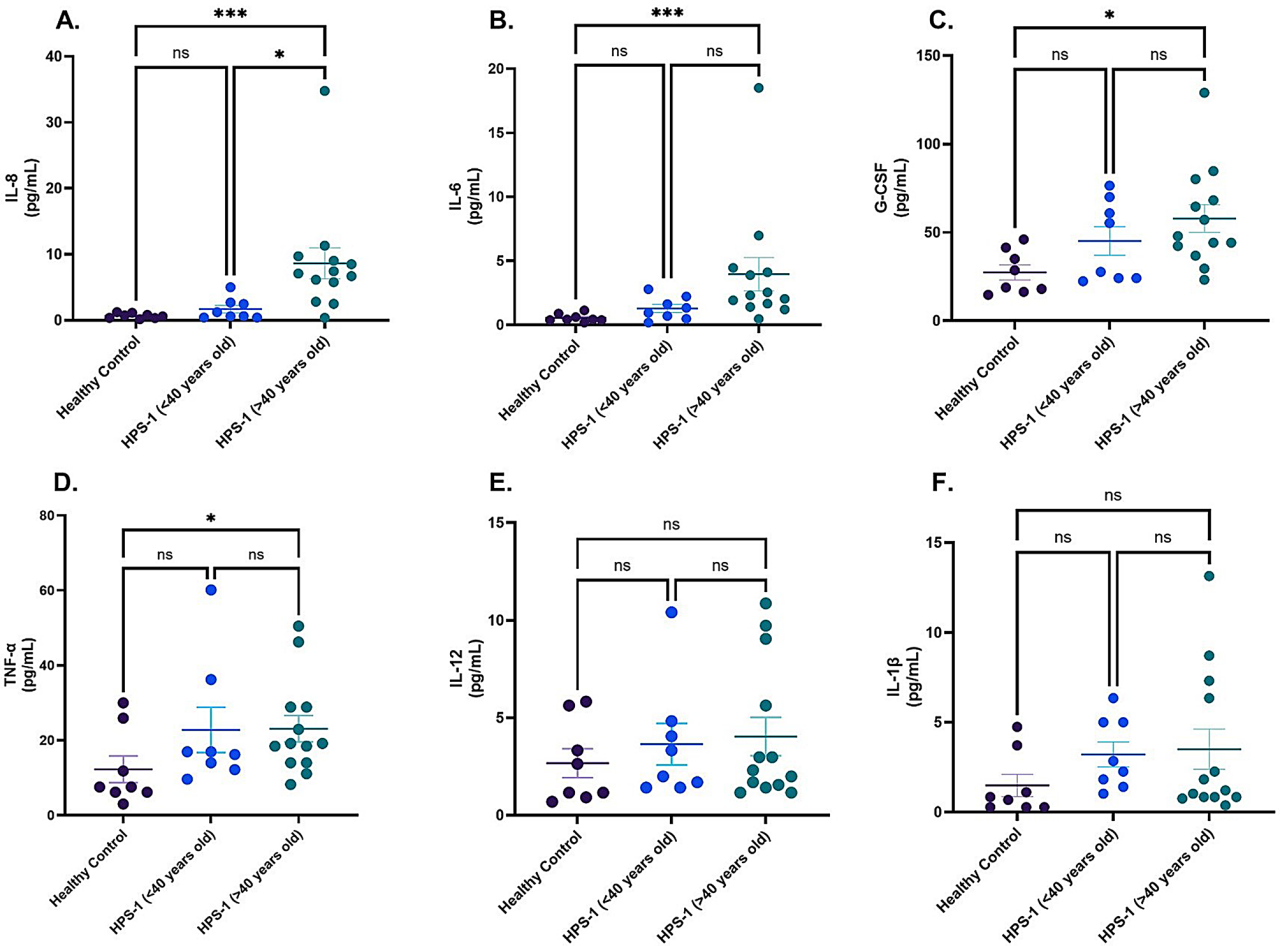
Levels of inflammatory markers in patients with HPS-1. The biomarkers shown are **A**) IL-8, **B**) IL-6, **C**) G-CSF, **D**) TNF-α, **E**) IL-12 P70, and **F**) IL-1β. Each data point represents an individual patient measurement within the group, with horizontal lines indicating the mean and error bars representing the standard error. The Kruskal-Wallis test was used to compare groups, with statistical significance indicated by asterisks: * for *p* < 0.05, ** for *p* < 0.01, and *** for *p* < 0.001

We performed a similar age stratification to assess circulating levels of IL-8, IL-6, G-CSF, TNF-α, IL-1β, and IL-12 P70 in patients with HPS-1 (*n* = 21). IL-8, a potent neutrophil-attracting chemokine, was significantly elevated in patients with HPS-1 over 40 years old (*n* = 13) compared to both the under 40 group (*n* = 8) (*P* < 0.05) and healthy controls (*n* = 8) (*P* < 0.001) Fig. 2A, suggesting enhanced neutrophil recruitment in older patients. IL-6 levels were also significantly increased in the over 40 group compared to healthy individuals (*P* < 0.001) Fig. 2B, indicating a stronger inflammatory response in this cohort. Similarly, G-CSF, a key regulator of neutrophil production and differentiation, was significantly elevated in HPS-1 patients over 40 years old compared to healthy controls (*P* < 0.05) Fig. 2C, further supporting increased neutrophil activity in older individuals. Additionally, TNF-α levels were significantly higher in individuals over 40 years of age compared to healthy controls (*P* < 0.05) Fig. 2D. In contrast, there were no statistically significant differences in the levels of IL-12 P70, and IL-1β between groups Figs. 2E and F.

To address the potential contribution of type 2 immunity to fibrosis development in HPS-1, we measured plasma levels of IL-4 and IL-13 in patients with HPS-1. Our results indicate that IL-4 levels were significantly higher in patients over 40 years old compared to those younger than 40 Figure S1A. While IL-13 levels showed a trend towards being higher in the older age group, this difference did not reach statistical significance Figure S1B.

### Neutrophil activation markers and anandamide were associated in patients with HPS-1 over 40

**Fig. 3:**
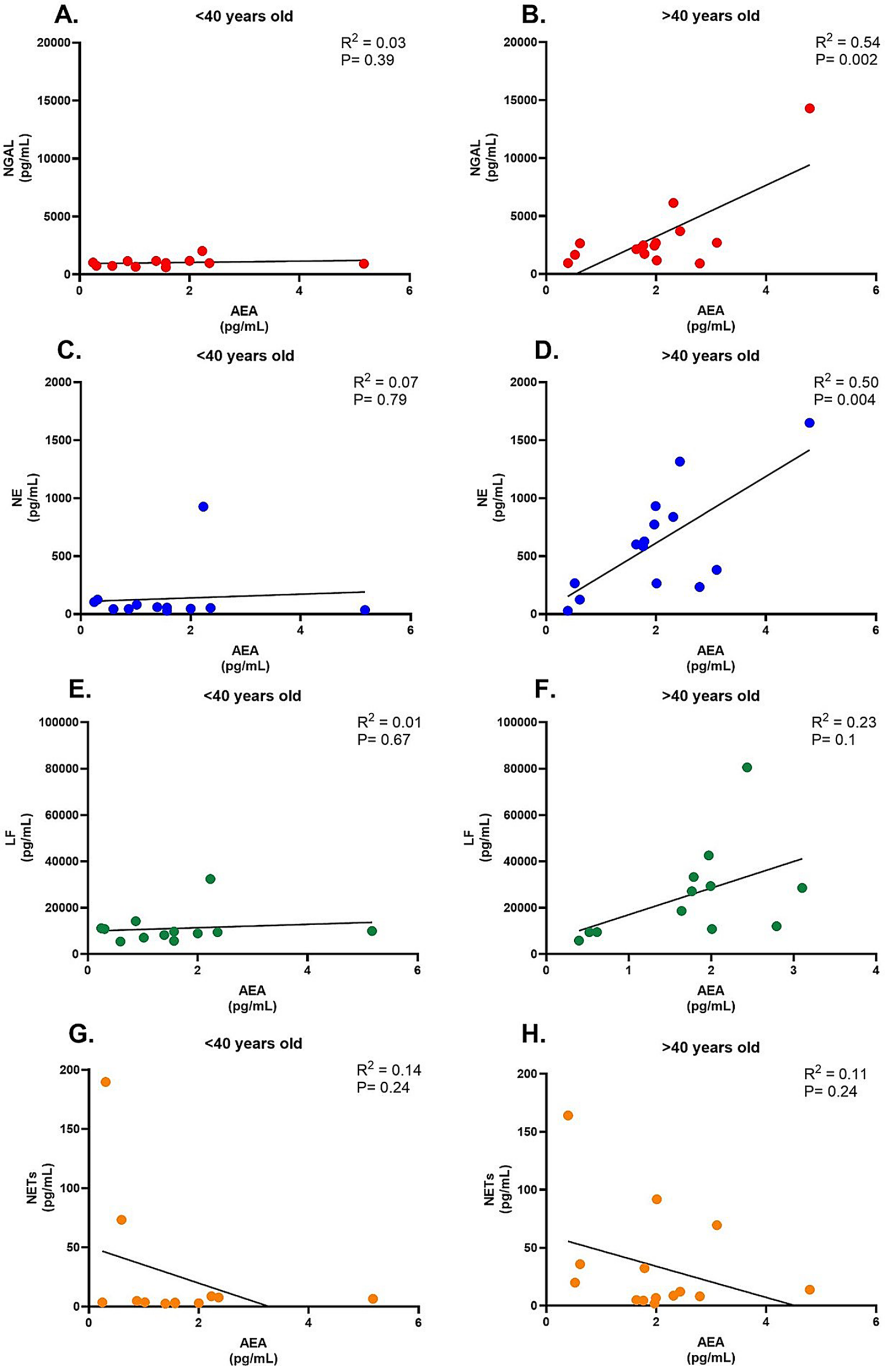
Association of circulating NETs and neutrophil granule-derived proteins with Anandamide (AEA) concentration in patients with HPS-1. Simple linear regression analysis showing the relationship between AEA (pg/mL) to the following biomarkers: (**A** and **B**) Neutrophil Gelatinase-Associated Lipocalin (NGAL), (**C** and **D**) Neutrophil Elastase (NE), (**E** and **F**) Lactoferrin (LF), and (**G** and **H**) Neutrophil Extracellular Traps (NETs). Each panel depicts a scatter plot with a fitted regression line. The coefficient of determination (R²) and the p-values are indicated in each plot

We did not observe a significant association between neutrophil markers and FVC% Figure S2 A–F. Interestingly, the only marker that showed the expected trend towards a negative association with FVC% was NETs, (R^2^ = 0.1090, *P* = 0.2489) Figure S2H. Since FVC may not effectively capture early fibrotic changes—given that it primarily measures lung volume rather than subtle fibrotic progression—we further evaluated FEV1/FVC%, focusing on ratios above 80% to exclude cases indicative of obstructive disease (typically seen with ratios below 80%). This analysis indicated a significant positive association between NE and FEV1/FVC (R^2^ = 0.3985, *P* = 0.0277) Figure S3D, while the other markers (NGAL, LF) Figure S3 B and F, showed a trend but no significant differences. Like FVC%, FEV1/FVC % showed an important trend towards a negative association with NETs, (R^2^ = 0.3290, *P* = 0.0512) Figure S3H. To address these limitations, we examined anandamide (AEA), a recently identified marker that may detect fibrotic changes before significant tissue injury [14]. We stratified our analyses by age groups—patients under 40 and those over 40 years old—to account for potential age-related onset of fibrosis in older individuals. Our results showed that in patients under 40, NGAL, levels did not significantly correlate with AEA (R² = 0.03, *P* = 0.39) Fig. 3A. However, in patients over 40, NGAL exhibited a significant positive relationship with AEA (R²= 0.54, *P* = 0.002) Fig. 3B. Similarly, NE levels were not significantly associated with AEA in the under 40 group (R² = 0.07, *P* = 0.79) Fig. 3C, while a positive association was observed in the over 40 group (R² = 0.50, *P* = 0.004) Fig. 3D. LF levels showed no significant association with AEA in patients under 40 (R² = 0.01, *P* = 0.67) Fig. 3E and only a weak, non-significant trend in those over 40 (R² = 0.23, *P* = 0.1) Fig. 3F. NETs demonstrated weak trends in both age groups, without significant associations in the under 40 group (R² = 0.14, *P* = 0.24) or the over 40 group (R² = 0.11, *P* = 0.24) Fig. 3G and H. Interestingly, NETs were the only marker to show a negative slope.

To further investigate markers associated with extracellular matrix remodeling and neutrophil-driven inflammation, we assessed circulating levels of MMP-7, MMP-8, and MMP-9 in patients with HPS-1 (*n* = 12). Consistent with the increased neutrophil activation observed in older patients, MMP-7, a well-established pro-fibrotic mediator and potential diagnostic biomarker for IPF, was significantly elevated in patients over 40 years old (*n* = 7) compared to healthy controls (*n* = 4) (*P* < 0.05) Figure S4A. This suggests a possible increase in extracellular matrix remodeling and epithelial injury with age. Similarly, MMP-8 levels were significantly higher in patients over 40 years old compared to those 40 years or younger (*P* < 0.05) Figure S4B, indicating enhanced neutrophil-driven inflammation and collagen degradation. However, MMP-9 levels did not show statistically significant differences, although a trend toward higher levels was noted in older patients compared to both younger patients and healthy controls Figure S4C.

### Age-Related differences in NETosis capacity in patients with HPS-1

**Fig. 4:**
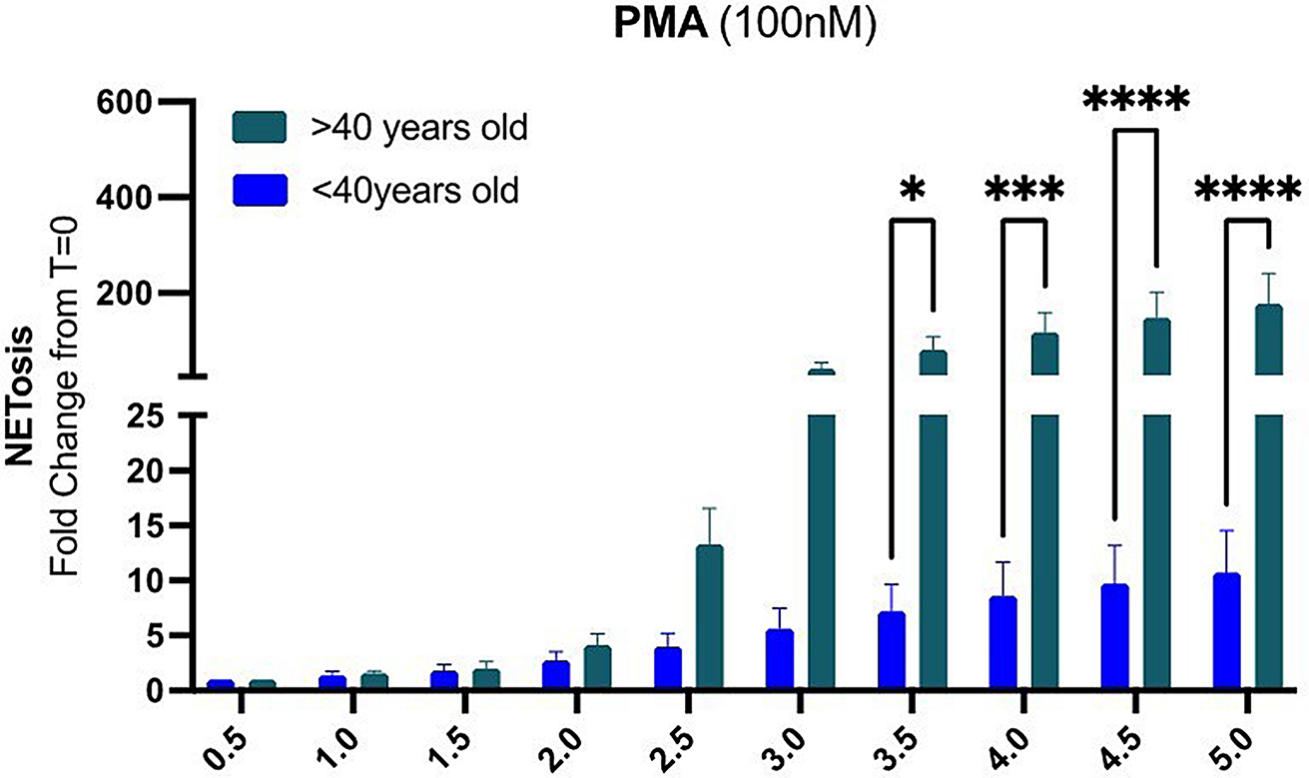
Age-Related Differences in NETosis Capacity in Patients with HPS-1. NETosis capacity of primary neutrophils isolated from patients under 40 years old (*n* = 4) and over 40 years old (*n* = 6) upon stimulation with 100 nM PMA. Data are presented as fold change from time 0, with bars representing mean ± standard error of the mean (SEM). Statistical analysis was performed using a two-way ANOVA with multiple comparisons. Significant differences are denoted as follows: *p < 0.05, **p < 0.01, ***p < 0.001

We performed an ex-vivo functional assay to evaluate the capacity of form NETs in response to PMA. We observed age-related differences in NETs formation between neutrophils from individuals under 40 (*n* = 4) and those over 40 years old (*n* = 6). NETs formation was minimal in both groups during the initial 2.5 h. However, after 2.5 h, NETs formation in neutrophils from individuals over 40 increases compared to the younger group, with differences becoming statistically significant (*P* < 0.05) after 3.5 h. In contrast, neutrophils from the under-40 group exhibited only a modest increase in NETs formation Fig. 4.

## Discussion

This study explores the involvement of neutrophil activation, specifically the circulating levels of NETs, granule proteins and cytokines in HPS-1. The findings suggest a potential association between neutrophil-derived mediators, such as NETs, NE, NGAL, and LF, and age, particularly in patients over 40 years old. Additionally, the assessment of these markers in relation to AEA, an early fibrosis marker, provides further insight into their potential relevance in the context of HPS-PF. Given the absence of targeted therapies for HPS-PF, these insights highlight the need to further investigate neutrophil-mediated pathways in disease progression. The distinct profile of neutrophil activation markers in HPS-1 aligns with existing literature on the fibrogenic role of NETs in chronic lung diseases, such as IPF [12, 15, 16]. Also, in previous studies with animal models, neutrophils exhibit altered trafficking and function in response to fibrosis, suggesting that neutrophils may become persistently activated and fail to undergo proper clearance in fibrotic tissue, thereby exacerbating inflammation and advancing fibrotic progression in HPS-PF [17]. Similarly, in our functional evaluation we observed neutrophil response increase with age in patients with HPS-1 with differences more noticeable over 40 years old.

Elevated NETs, observed in the over-40 HPS-1 cohort, suggest that neutrophil activation may intensify with age, particularly the inflammatory signals [18]. This is consistent with previous studies showing that aging affects NETosis by increasing NETs production but reducing their antimicrobial effectiveness [19]. The study showed that instead of responding primarily to pathogens, NETs in older individuals form in response to inflammatory signals. Additionally, NETs from elderly people contain larger DNA structures than those from younger adults, which may further impact their functionality. This age-related increase in NETs and granule-derived proteins, which are known to drive fibrosis through fibroblast activation and epithelial cell injury [20], suggests a potential pathway of progressive lung damage in HPS-PF that warrants further mechanistic studies.

Our study also reveals that specific cytokines, notably IL-8 and IL-6, are significantly elevated in older patients with HPS-1, proposing enhanced neutrophil recruitment and activation in this age group. IL-8, a potent neutrophil-attracting chemokine [21], was significantly elevated in patients over 40, supporting its role in recruiting neutrophils to the lung, where they may exacerbate tissue damage and fibrosis. This cytokine has been shown to be upregulated in HPS-PF [22]. Also, in IPF, serum IL-8 levels were significantly higher compared to healthy controls, with positive correlations observed between serum IL-8 and both the percentage of neutrophils in BAL and indicators of disease severity, such as impaired lung function [23]. Similarly, elevated IL-6, TNF-α, G-CSF levels in older patients suggest increased systemic inflammation and neutrophil differentiation, further contributing to the escalation of neutrophil activity and NETs formation in patients over 40 years old.

Given the potential confounding effect of aging on neutrophil activation markers, we performed a linear regression analysis to evaluate the association between biomarker levels and age in healthy controls. Interestingly, control subjects demonstrated a negative association between age and neutrophil activation markers (NETs, NE, N-GAL, LF) as well as inflammatory cytokines (IL-8, IL-6, G-CSF, TNFα), whereas patients with HPS-1 exhibited a positive association Figure S5 and S6. These findings propose that neutrophil activation observed in HPS-1 is not due to normal aging processes but may instead reflect accelerated or intensified age-associated neutrophil activation driven by the HPS-1 mutation. This aligns with prior studies highlighting age-related changes in HPS-1 models [24], further supporting the hypothesis that genetic factors in HPS-1 patients may amplify inflammatory pathways underlying disease progression.

To assess the potential involvement of type 2 immunity in HPS-1 disease, circulating IL-4 and IL-13 levels were evaluated. IL-4 was significantly elevated in patients over 40 years old compared to younger patients, whereas IL-13 showed only a non-significant upward trend. These findings align with evidence suggesting that type 2 cytokines, perhaps released ILC2s play a role in HPS-PF, aligning with previous studies [25]​.

AEA, an endogenous cannabinoid, proven to be an early indicator of fibrotic progression in HPS-PF [14]. In patients over 40, AEA levels were positively associated with neutrophil activation markers such as NE and NGAL, suggesting its role as an early biomarker of HPS-PF that may detect disease activity before conventional lung function metrics like FVC show significant decline. Interestingly, these associations were not observed in patients under 40, indicating that neutrophil activation and AEA levels may become more pronounced with age. Further investigation of this relationship could provide valuable insights for future clinical trials targeting neutrophil-mediated processes to delay fibrosis progression. Additionally, circulating matrix metalloproteinases (MMP-7, MMP-8, and MMP-9) were assessed as exploratory markers of fibrosis. Elevated MMP-7 and MMP-8 levels in older patients suggest increased extracellular matrix remodeling and neutrophil-driven inflammation, consistent with previous findings in both HPS-PF and IPF, where MMPs have been implicated in disease progression [26, 27]. These results further support the role of age-related immune dysregulation in fibrosis development in HPS-1.

Intriguingly, NETs—DNA-rich structures released by neutrophils—exhibited a negative association with AEA levels in circulating blood, yet studies of HPS-1 lung tissue reveal significant local NETs activity, as evidenced by the presence of citrullinated histone H3 (citH3) and myeloperoxidase (MPO) in fibrotic lungs [13]. These findings suggest that NETs contribute predominantly to fibrosis through localized lung activity rather than via circulating blood levels. NETs in the lungs adhere more firmly to tissue structures due to their DNA-rich composition, as has been shown in cancer [28], driving persistent inflammation and promoting fibrotic changes. In contrast, soluble neutrophil activation markers like NE and NGAL diffuse into the bloodstream, showing stronger associations with circulating AEA levels and serving as systemic indicators of neutrophil activity.

Although more pronounced, age-related neutrophil activation is not unique to HPS, we hypothesize the following may serve as a plausible explanation for its contribution to fibrosis in these patients. HPS is characterized by dysfunction of lysosome-related organelles [29], this can impair key cellular processes such as phagocytosis and autophagy [30]. This dysfunction may explain the localized accumulation of NETs in the lungs of patients with HPS-1. Normally, macrophages will degrade NETs through extracellular pre-digestion followed by uptake and lysosomal degradation [31]. However, defective lysosomal pathways in macrophages in HPS-1 may limit their ability to effectively process NETs, leading to their persistence in lung tissue. Consistent with this idea, researchers observed association of NETs markers, such as citrullinated histone H3 (citH3) and myeloperoxidase (MPO), with macrophages in explanted lungs from patients with HPS-PF [13]. This could explain why comorbid conditions or environmental exposures, will enhance infiltration of neutrophils into the lungs could act as accelerants of HPS-PF, inducing an earlier or more severe fibrotic response. Moreover, it is consistent a higher sensitivity to bleomycin-induced PF in a naturally occurring HPS-1 mouse model [32].

This study has several limitations. First, the weak associations between FVC% and neutrophil markers highlight the limitations of FVC in detecting early fibrotic changes, as it primarily measures lung volume and may miss subtle parenchymal damage [33]. This emphasizes the need for additional clinical assessment (e.g. DLCO) or biomarkers like AEA, detect early fibrosis. Second, the small number of patients with confirmed HPS-PF limits our ability to draw definitive conclusions about the role of neutrophil activation in fibrosis progression. Larger, longitudinal studies are needed to validate these findings and assess the predictive value of neutrophil markers. Similarly, the observed increase in NETosis capacity in older individuals with HPS-1 should be interpreted with caution due to the small sample size. Additionally, reliance on retrospective CT scan reports for fibrosis diagnosis may introduce variability. Prospective studies with standardized imaging and functional assessments would strengthen these findings. Lastly, while we evaluated circulating markers of neutrophil activation, they may not fully reflect localized lung inflammation. Future studies incorporating bronchoalveolar lavage or lung biopsy samples could provide deeper insights.

Despite these limitations, this study provides evidence of age-related neutrophil activation in HPS-1, with elevated levels of neutrophil-derived mediators and inflammatory cytokines observed in older patients. While these findings suggest a potential link between neutrophil activation, inflammation, and fibrotic processes, further research is needed to clarify the underlying mechanisms and their role in disease progression. Understanding these processes could inform the development of targeted therapies and novel biomarkers to improve early detection and management of HPS-PF.

## Electronic supplementary material

Below is the link to the electronic supplementary material.

Supplementary Material 1

## Data Availability

All data generated or analyzed during this study are included in this published article and its supplementary information files.
