# Supplementary material for: Age-related neutrophil activation in Hermansky-Pudlak Syndrome Type-1
Source: Orphanet J Rare Dis. 2025 May 12;20:226. doi: 10.1186/s13023-025-03758-5 (PMC12067913; doi:10.1186/s13023-025-03758-5)
Supplement: Supplementary file 1 — Supplementary Material 1 [file 13023_2025_3758_MOESM1_ESM.docx]

**Age-related neutrophil activation in Hermansky-Pudlak Syndrome Type 1.**

Lourdes Marinna Caro-Rivera^1^, Sonya Malavez-Cajigas^1^, Mercedes Lacourt-Ventura^1^, Andrea P. Rivera-Torres^1^, Dorca E. Marcano-Jiménez^1^, Pablo López-Colon ^1^, José Muñiz-Hernández^2^, Enid Rivera-Jiménez^1^, Mónica Egozcue-Dionisi^3^, Rosa Román-Carlo^1^, Wilfredo De Jesús-Rojas^1^, and Marcos J. Ramos- Benítez^1^*

1. Department of Basic Sciences, Ponce Health Sciences University and Ponce Research Institute, Ponce, PR.
2. Department of Medicine, San Juan Bautista School of Medicine, Caguas, PR.
3. Department of Pediatrics, University of Puerto Rico, Medical Sciences Campus, San Juan, PR.

*Corresponding author

Marcos J. Ramos- Benítez, [mjramos@psm.edu](mailto:mjramos@psm.edu)

**Supplementary Figures**

**
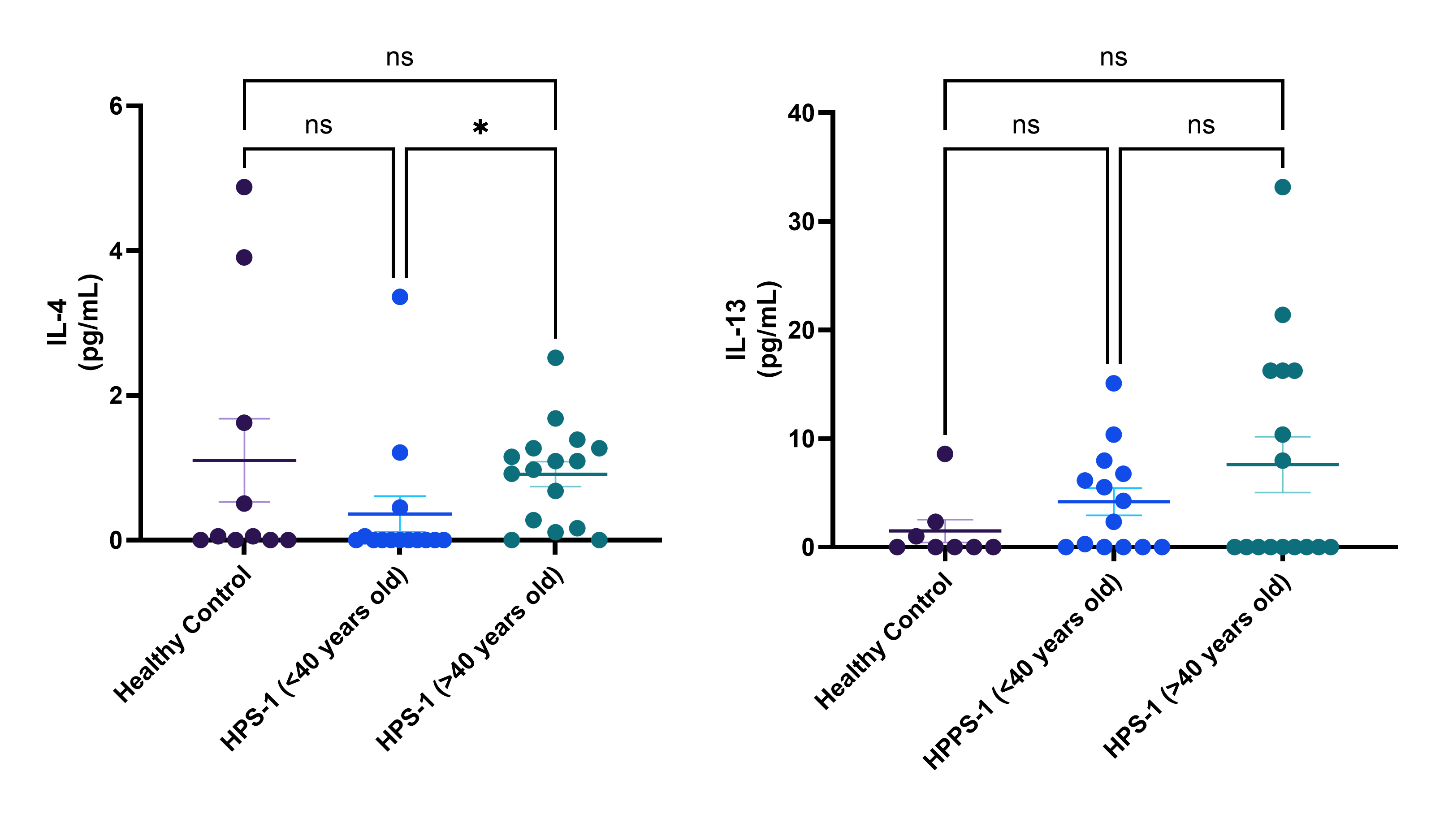
**

**B**

**A**

**Figure S1. Levels of IL-4 and IL-13 in patients with HPS-1.** The biomarkers shown are A) IL-4, and B) IL-13. Each data point represents an individual patient measurement within the group, with horizontal lines indicating the mean and error bars representing the standard error. The Kruskal-Wallis test was used to compare groups, with statistical significance indicated by asterisks: * for p < 0.05, ** for p < 0.01, and *** for p < 0.001.


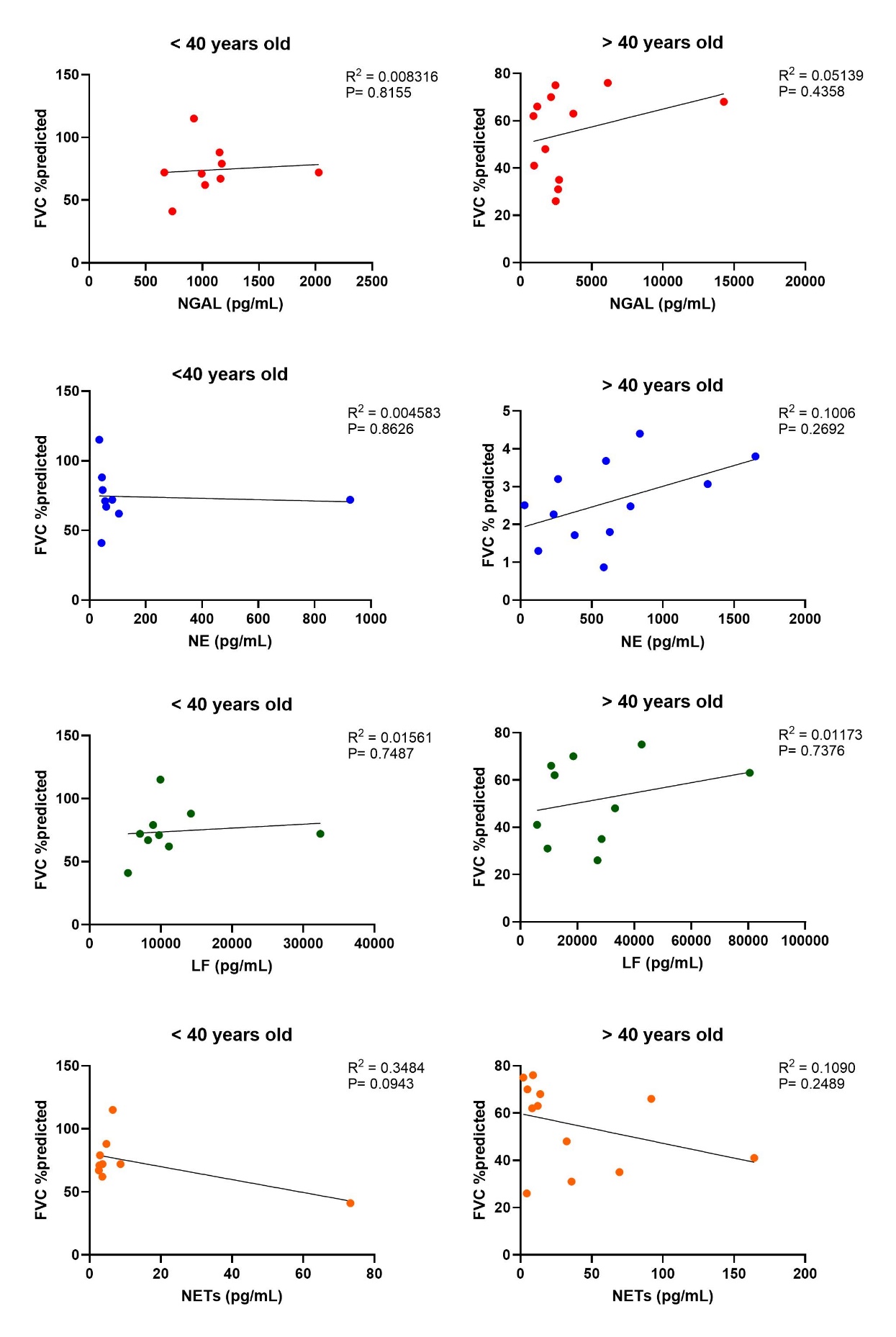


**A**

**B**

**C**

**D**

**E**

**F**

**G**

**H**

**Figure S2. Association of circulating NETs and neutrophil granule-derived proteins with FVC % in patients with HPS-1.** Simple linear regression analysis showing the relationship between FVC % predicted to the following biomarkers: (A and B) Neutrophil Gelatinase-Associated Lipocalin (NGAL), (C and D) Neutrophil Elastase (NE), (E and F) Lactoferrin (LF), and (G and H) Neutrophil Extracellular Traps (NETs). Each panel depicts a scatter plot with a fitted regression line. The coefficient of determination (R²) and the p-values are indicated in each plot, demonstrating a significant.


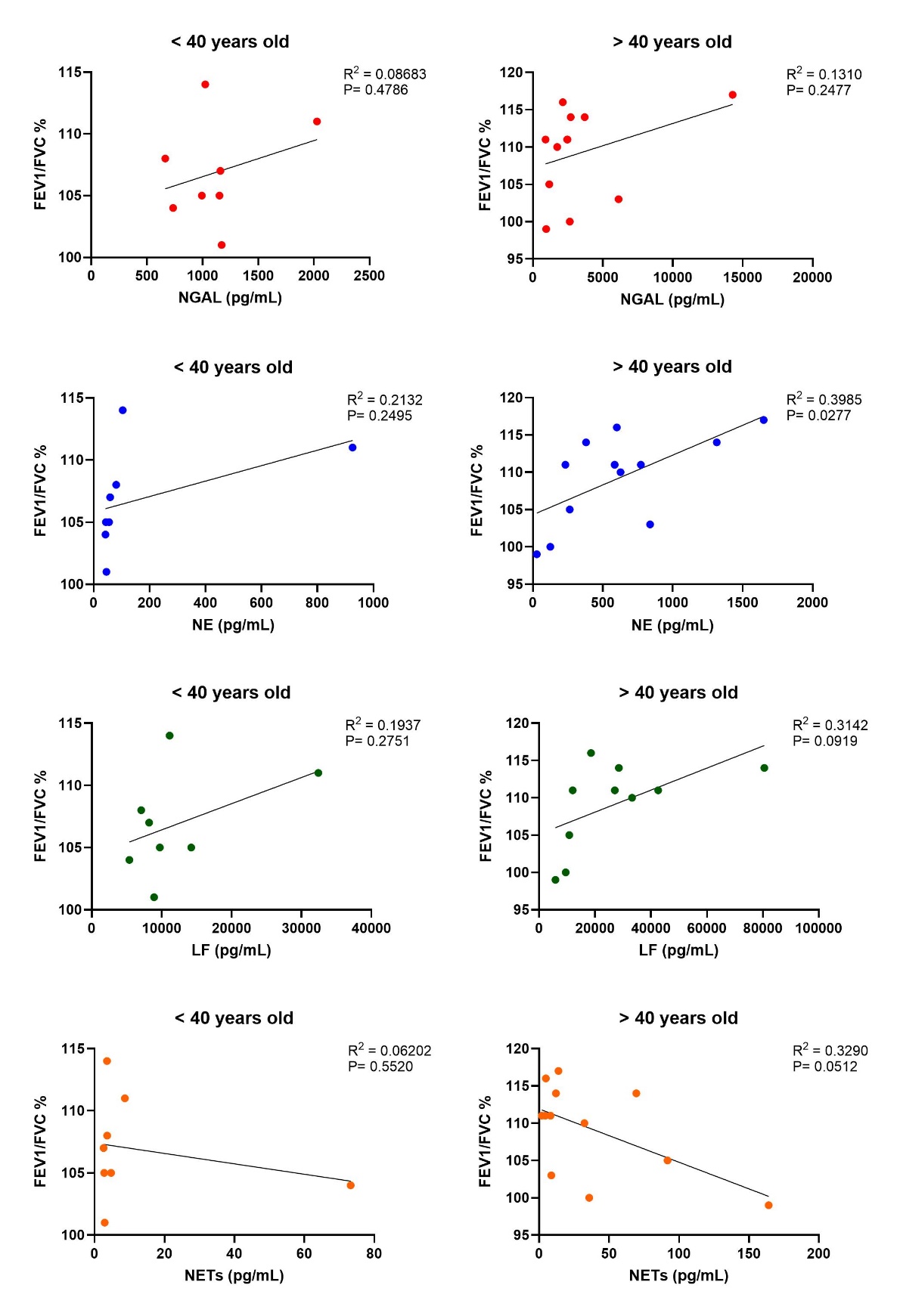


**A**

**B**

**C**

**D**

**E**

**F**

**G**

**H**

**Figure S3. Association of circulating NETs and neutrophil granule-derived proteins with FEV1/FVC % in patients with HPS-1.** Simple linear regression analysis showing the relationship between FEV1/FVC % to the following biomarkers: (A and B) Neutrophil Gelatinase-Associated Lipocalin (NGAL), (C and D) Neutrophil Elastase (NE), (E and F) Lactoferrin (LF), and (G and H) Neutrophil Extracellular Traps (NETs). Each panel depicts a scatter plot with a fitted regression line. The coefficient of determination (R²) and the p-values are indicated in each plot, demonstrating a significant.


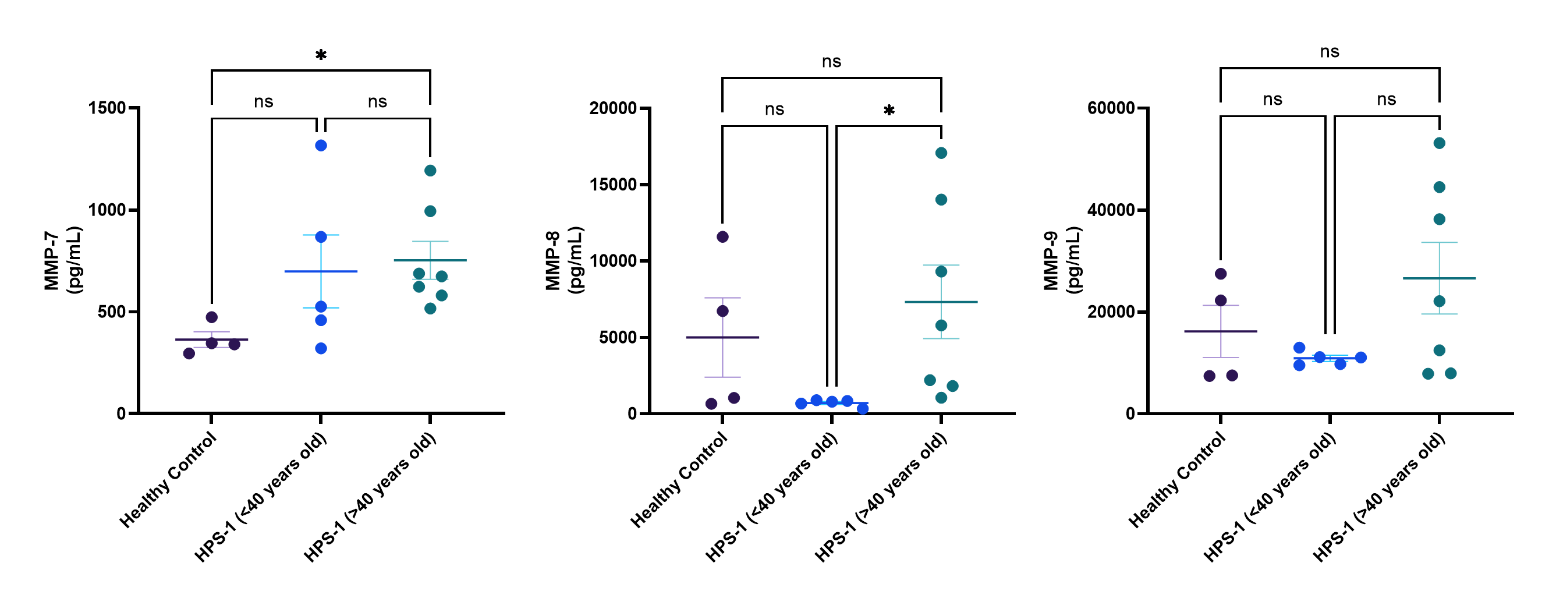


**C**

**B**

**A**

**Figure S4. Levels MMP-7, 8 and 9 in patients with HPS-1.** The biomarkers shown are A) MMP-7, B) MMP-8, and C) MMP-9. Each data point represents an individual patient measurement within the group, with horizontal lines indicating the mean and error bars representing the standard error. The Kruskal-Wallis test was used to compare groups, with statistical significance indicated by asterisks: * for p < 0.05, ** for p < 0.01, and *** for p < 0.001.


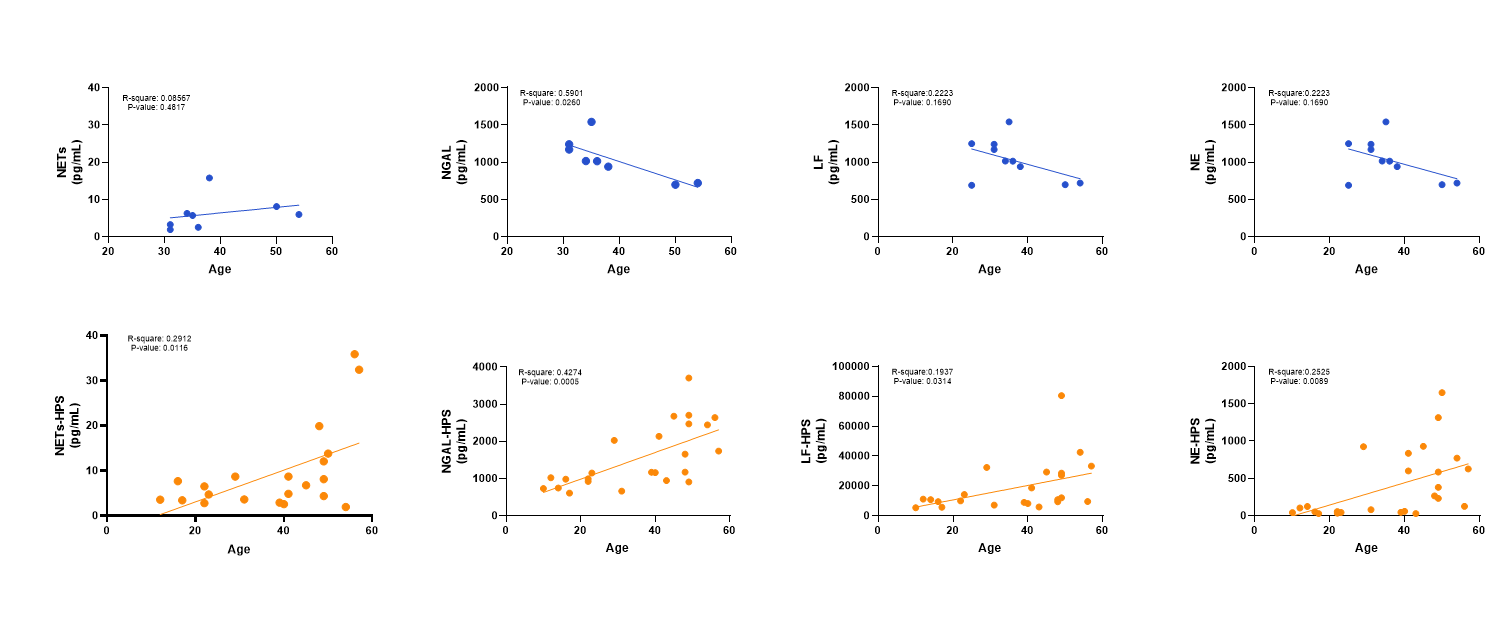


**A**

**B**

**C**

**D**

**H**

**G**

**F**

**E**

**Figure S5. Association of circulating NETosis markers and age in patients with HPS-1.** Simple linear regression analysis shows the relationship between the following NETosis biomarkers and age: (A and B) NETs, (C and D) N-GAL, (E and F) LF, and (G and H) NE. Each panel depicts a scatter plot with a fitted regression line. The coefficient of determination (R²) and the p-values are indicated in each plot.


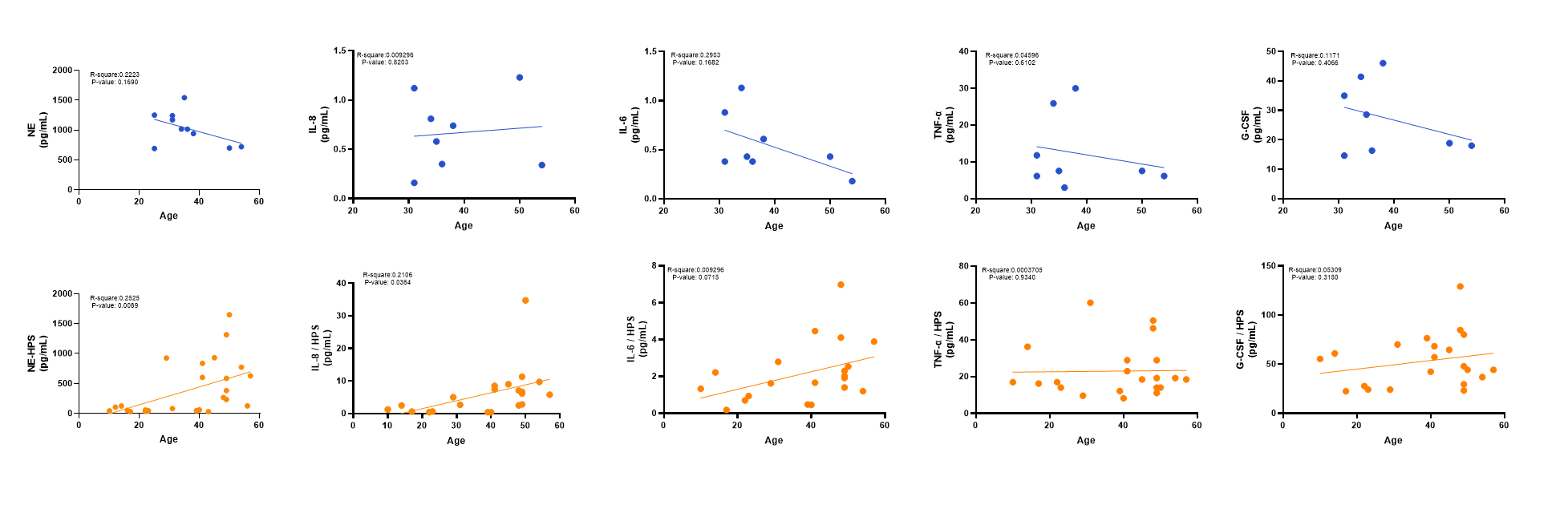


**F**

**B**

**A**

**C**

**D**

**E**

**G**

**H**

**Figure S6. Association of circulating inflammatory biomarkers and age in patients with HPS-1.** Simple linear regression analysis shows the relationship between the following biomarkers and age: (A and B) IL-8, (C and D) IL-6, (E and F) TNF-α, and (G and H) G-CSF. Each panel depicts a scatter plot with a fitted regression line. The coefficient of determination (R²) and the p-values are indicated in each plot.
